# The Therapeutic Potential of Novel Carnosine Formulations: Perspectives for Drug Development

**DOI:** 10.3390/ph16060778

**Published:** 2023-05-23

**Authors:** Angela Bonaccorso, Anna Privitera, Margherita Grasso, Sonya Salamone, Claudia Carbone, Rosario Pignatello, Teresa Musumeci, Filippo Caraci, Giuseppe Caruso

**Affiliations:** 1 Department of Drug and Health Sciences, University of Catania, 95125 Catania, Italy; 2 NANOMED–Research Centre for Nanomedicine and Pharmaceutical Nanotechnology, University of Catania, 95125 Catania, Italy; 3 Department of Biomedical and Biotechnological Sciences, University of Catania, 95123 Catania, Italy; 4 Unit of Neuropharmacology and Translational Neurosciences, Oasi Research Institute-IRCCS, 94018 Troina, Italy

**Keywords:** carnosine, drug development, drug delivery, derivatives, conjugates, vesicular systems, nanoparticles

## Abstract

Carnosine (beta-alanyl-L-histidine) is an endogenous dipeptide synthesized via the activity of the ATP-dependent enzyme carnosine synthetase 1 and can be found at a very high concentration in tissues with a high metabolic rate, including muscles (up to 20 mM) and brain (up to 5 mM). Because of its well-demonstrated multimodal pharmacodynamic profile, which includes anti-aggregant, antioxidant, and anti-inflammatory activities, as well as its ability to modulate the energy metabolism status in immune cells, this dipeptide has been investigated in numerous experimental models of diseases, including Alzheimer’s disease, and at a clinical level. The main limit for the therapeutic use of carnosine is related to its rapid hydrolysis exerted by carnosinases, especially at the plasma level, reason why the development of new strategies, including the chemical modification of carnosine or its vehiculation into innovative drug delivery systems (DDS), aiming at increasing its bioavailability and/or at facilitating the site-specific transport to different tissues, is of utmost importance. In the present review, after a description of carnosine structure, biological activities, administration routes, and metabolism, we focused on different DDS, including vesicular systems and metallic nanoparticles, as well as on possible chemical derivatization strategies related to carnosine. In particular, a basic description of the DDS employed or the derivatization/conjugation applied to obtain carnosine formulations, followed by the possible mechanism of action, is given. To the best of our knowledge, this is the first review that includes all the new formulations of carnosine (DDS and derivatives), allowing a decrease or complete prevention of the hydrolysis of this dipeptide exerted by carnosinases, the simultaneous blood–brain barrier crossing, the maintenance or enhancement of carnosine biological activity, and the site-specific transport to different tissues, which then offers perspectives for the development of new drugs.

## 1. Introduction

The dipeptide carnosine (beta-alanyl-L-histidine) was discovered more than 100 years ago during a study carried out by Gulewitsch and Amiradžibi, both working at the Laboratorium der Universität Charkow (Ukraine), in which a meat extract was analyzed [1]. At the end of the analysis, different unknown nitrogen-containing compounds, including carnosine, were obtained. Based on the sample analyzed (minced meat), the molecule was named “carnosine”, coming from the Latin *caro*, *carnis* (meat).

The synthesis of carnosine starting from β-alanine and L-histidine is related to the activity of the enzyme carnosine synthetase 1 (CARNS1). Carnosine is physiologically present in different mammalian tissues, with the highest tissue concentrations (millimolar order) observed in cardiac and skeletal muscles as well as at the central nervous system (CNS) level [1].

Carnosine possesses a well-known multimodal mechanism of action, including anti-aggregant, anti-inflammatory, and antioxidant properties [2,3,4,5], and has also shown the ability to enhance both antioxidant machinery [6] and energy metabolism [7,8,9] in different cell types, reasons why researchers have been encouraged to investigate its therapeutic potential in numerous multifactorial disorders such as Alzheimer’s disease [10,11,12], depression [13,14], and Parkinson’s disease [15,16].

In spite of high expectations, an important limit for carnosine therapeutic application is the reduction of its bioavailability due to degradation. Indeed, carnosine is cleaved by two human carnosinases, the serum-circulating carnosine dipeptidase 1 (CNDP1) and the cytosolic carnosine dipeptidase 2 (CNDP2), which is known to strongly reduce the bioavailability of carnosine [17,18]. For this reason, during the last two decades, different research groups have been working on the development of new approaches (e.g., drug delivery systems (DDS)) and new pharmacological formulations of carnosine in order to protect carnosine against carnosinases’ degradation, then improving its bioavailability, and/or its ability to reach a specific target [19]. Based on the above, it also becomes clear the substantial heterogeneity regarding the route of carnosine administration in in vivo preclinical studies, where the oral administration through drinking water and the intraperitoneal (i.p.) injection represent the most widely employed [18]. One administration route that is attracting a lot of attention is the intranasal one [20], a route that might bypass the blood–brain barrier (BBB) and the first-pass metabolism [21,22]. Alternative and innovative approaches able to increase carnosine delivery include the use of vesicular (nanoliposomes, niosomes, and polymerosomes) and nanoparticulate systems. Nanovesicle systems are commonly used in the prognosis, diagnosis, and treatment of premalignant gastrointestinal tumors [23], while exosomes, cell-derived nanovesicles with a diameter ranging from 30 to 150 nm, have been considered in cancer therapy [24]. Niosomal formulations can be coupled to specific ligands able to be recognized by the BBB transporters [25]. Recently, nanoformulations, including nanovesicles, solid-lipid nanoparticles (NPs), nanoemulsions, and polymeric NPs, have shown promising results in the improvement of both efficacy and bioavailability of molecules of interest, as observed in the case of vitamin E [26]. Nanocapsules, composed of an inner liquid core and surrounded by a polymeric wall, are considered excellent carriers for a wide range of active pharmaceutical molecules [27], while nanoparticulate systems offer, among many, the advantage of improving the oral bioavailability of hydrophobic drugs [28].

The aim of this study was to explore the therapeutic potential of novel carnosine formulations and the perspectives for drug development, providing a narrative and critical analysis of the existing literature. We specifically focused on vesicular and nanoparticulate systems as well as on derivatives/conjugates able to increase carnosine activities, its stability to carnosinases, and/or facilitate the site-specific transport to different tissues.

## 2. Carnosine Structure, Biological Activities, Administration Routes, and Metabolism

**Figure 1:**
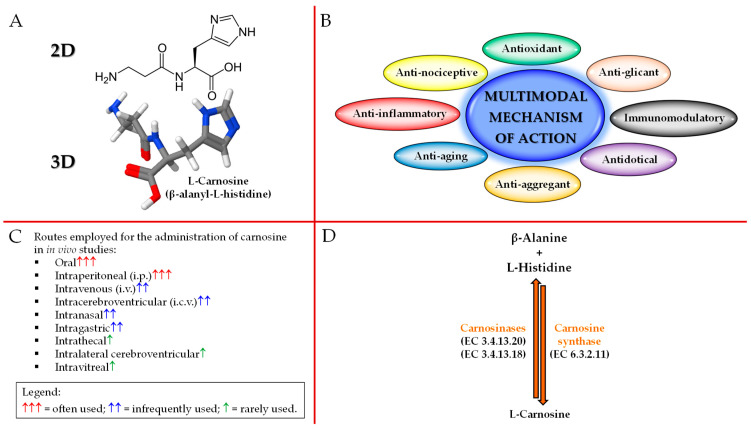
Schematic illustration of carnosine: (**A**) 2D and 3D structure, (**B**) biological activities, (**C**) administration routes, and (**D**) metabolism.

As previously mentioned, carnosine is a dipeptide composed of β-alanine and L-histidine that are joined by CARNS1 enzyme (EC 6.3.2.11) [29,30] (Figure 1A,D).

With regards to the origin of the two amino acids forming carnosine, it is very different. In fact, β-alanine is synthesized at the liver level, primarily through uracil and thymine degradation [31], while L-histidine is an essential amino acid, thus not synthesized de novo in humans, that has to be ingested through the diet [32]. It is worth mentioning that, in mammals, β-alanine is used essentially for carnosine synthesis, while the remaining part is subjected to different metabolic pathways, including degradation and transamination.

Carnosine is able to exert numerous biological and physiological roles. In fact, this naturally occurring dipeptide possesses a multimodal mechanism of action (Figure 1B). It is important to underline that despite its “preferential” localization (skeletal/cardiac muscles and brain), carnosine has shown the ability to exert biological activities in numerous and very different tissues. Since approximately 99% of carnosine can be found in muscle tissue [2], numerous studies have investigated the physiological activities of this dipeptide in muscles as well as the athletic benefits coming from its (or β-alanine precursor) supplementation. In addition to the well-demonstrated activities at muscle level, where carnosine has shown to favor muscle lactic acid detoxification and act as intramyocellular mobile buffer [33], also improving cytoplasmic Ca^2+^-H^+^ exchange/handling [34] and muscle contraction [35], mechanical work production (the so called “Severin’s phenomenon”) [36], muscle relaxation rates, as well as endurance exercise [37,38,39,40,41,42,43], carnosine can modulate energy metabolism in macrophages and microglia by restoring and/or enhancing the basal conditions (e.g., high-energy triphosphates and nicotinic coenzymes) [7,8,9,44], act as a neurotransmitter [45], regulate the activity of stem cells [46], modulate glucose metabolism, increasing fasting insulin levels and insulin resistance in non-diabetic overweight and obese individuals [47], enhance the degradation and/or scavenging of nitric oxide (NO) and related species [48,49,50], promote wound healing [51], exert anti-glycan and anti-aging activities [52,53], regulate osmotic pressure [54], modulate glutamate transport and production/metabolism at brain level [55], and interact with and chelate transition metals [56,57].

As described above, carnosine possesses several activities, but numerous research studies are still investigating its “additional” physiological roles. In this regard, in vivo studies employing transgenic models have been carried out. As recently described by Eckhardt and collaborators, mice knock-out (KO) for CARNS1 are characterized by reduced olfactory sensitivity [58]; in particular, the authors demonstrated that the absence of carnosine does not impair olfactory function in young CARNS1^−/−^ mice, but does in aging CARNS1^−/−^ mice, while there was an age-dependent decline in the number of olfactory receptor neurons in CARNS1^−/−^ that was not observed in wild-type mice, suggesting that carnosine is not essential for information processing in the olfactory signaling but plays a role in the long-term protection of olfactory receptor neurons. In a different study conducted by Wang-Eckhardt et al., the absence of carnosine synthesis does not significantly modulate the carbonylation of proteins, and the same applies to the formation of advanced lipoxidation end products in different tissues/organs such as muscles and brain [59].

Despite the above-described multitude of activities, the therapeutic potential of carnosine is often mitigated by its massive degradation into its constituting amino acids exerted by CNDP1 (EC 3.4.13.20; sieric) and CNDP2 (EC 3.4.13.18; cytosolic) enzymes [42,43,44] (Figure 1D), both part of the M20 metalloprotease family. In greater detail, CNDP1 specifically degrades carnosine and its GABA analog (homocarnosine) [42], while CNDP2, a non-specific dipeptidase ubiquitously expressed in human tissues, degrades carnosine as well as other dipeptides, but instead, it is not able to degrade homocarnosine [43]. As a consequence of what described above, with the aim to deliver carnosine to different districts/tissues, also trying to protect it from enzyme degradation, researchers have employed a wide range of administration routes including oral, i.p., intravenous (i.v.), intracerebroventricular (i.c.v.), intranasal, intragastric, intrathecal, intralateral cerebroventricular, and intravitreal [18] (Figure 1C).

## 3. Drug Delivery Systems

The term “drug delivery system” (DDS) refers to a formulation that enables the introduction of a therapeutic or diagnostic molecule in the body and improves its effectiveness and safety by controlling the rate, time, and site of drug release after administration [60].

**Figure 2:**
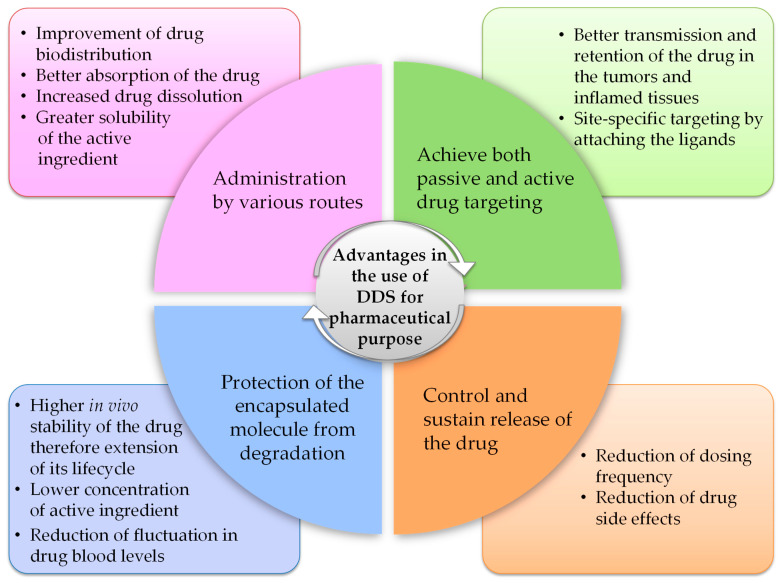
Advantages in the use of DDS for pharmaceutical purposes.

The first generation of DDS entered the market in the early 1950s and consisted of pharmaceutical formulations able to prolong drug activity and reduce dosing frequency [61]. DDS belonging to the “macroscopic scale era” (1950–1980) promoted drug release through dissolution, diffusion, osmosis, and ion exchange-based mechanisms, to produce systems that exhibited zero-order release rates, thus ensuring a constant drug plasma concentration (i.e., ophthalmic insert). The second generation of DDS (1980–2010) included both micro- (~1980s) and nano-sized (~1990–2000s) DDS as well as “smart” DDS technologies, the latter referring to systems developed to enable drug delivery in response to external stimuli, such as pH or temperature changes. This second generation is known as the “micro- and nano-scale era” and includes different DDS designed to promote a sustained and site-specific drug release (i.e., PEGylated DDS). The third generation of DDS (2010–present), defined as the “nanoscale era”, is based on DDS modulation to overcome physico-chemical and biological barriers. Active targeting has become a major focus (i.e., DDS targeted by monoclonal antibodies or cell membrane receptor ligands [62]) as well as understanding how they behave in vivo. Drug delivery research collects systems with different properties that can be classified based on their structure and composition into three main categories: vesicular (i.e., liposomes, niosomes, transferosomes, ethosomes, phytosomes, and polymerosomes), particulates (i.e., lipidic, polymeric and metallic NPs, nanogel, and nanocrystals), and supramolecular (i.e., cyclodextrins, micro-conjugates, bio-conjugates) systems. All these systems present different features and benefits (Figure 2), because the physico-chemical and pharmacokinetic properties of the entrapped drug are temporarily masked by those of the carrier.

The selection of DDS is strictly related to the properties of the molecule that has to be delivered to the target site, as well as to the selected route of administration.

To date, the literature suggests that since 2000 different types of DDS have been designed to deliver carnosine [19]. In the following paragraph, an overview of the general features of DDS currently exploited for carnosine delivery has been detailed, while in Table 1, their main advantages and limitations were reported.

### 3.1. Vesicular Systems: From Liposomes to Polymerosomes

Vesicular systems are highly ordered assemblies comprising one or multiple concentric bilayers formulated as an outcome of the self-assembling of amphiphilic molecules in water.

**Figure 3:**
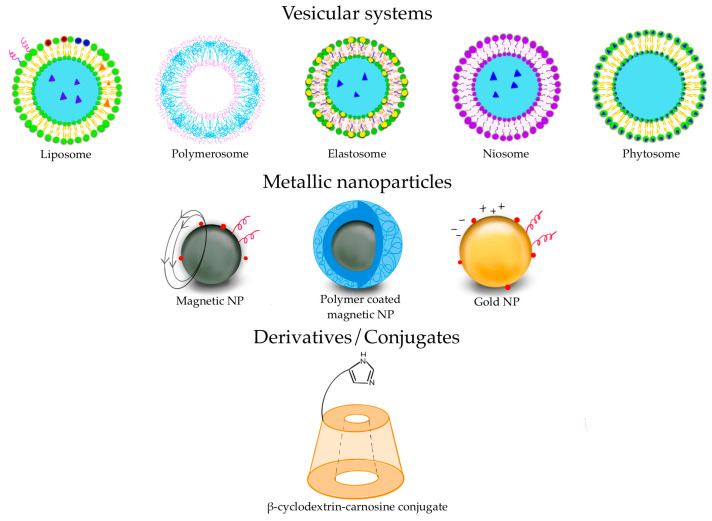
Some of the DDS used for pharmaceutical purposes.

Various types of vesicular systems, such as liposomes, polymerosomes, elastosomes, niosomes, and phytosomes can be included in this category of DDS [63,64] (Figure 3).

**Figure 4:**
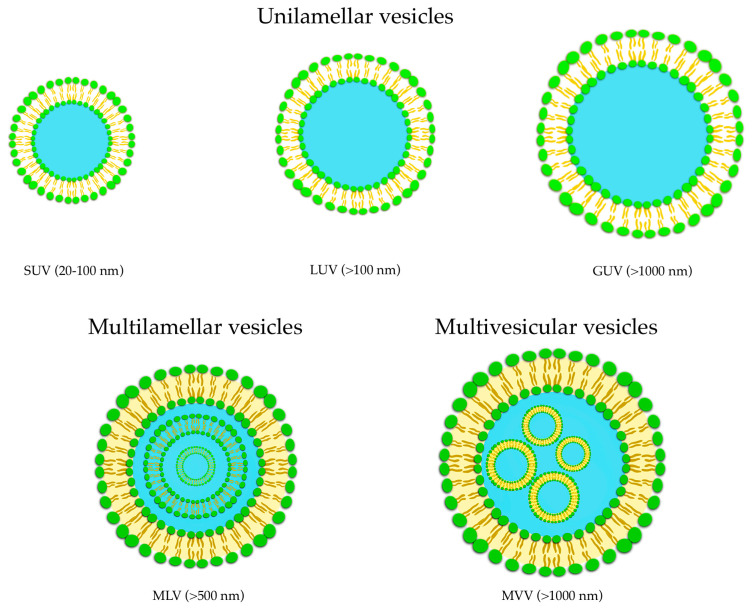
Classification of liposomes: unilamellar (ULV), multilamellar (MLV), and multivesicular (MVV) vesicles.

Their applications can be found in different fields such as dermatology, immunology, eye disorders, brain targeting, infective diseases, and tumor therapy, and they have also been considered as vaccine adjuvant [65]. Liposomes belong to this DDS class and can be described as spherical vesicles in which one or more lipid bilayer(s) entraps an aqueous volume, formed by self-assembly of amphiphilic lipid molecules, with the polar head groups oriented to the inner and the non-polar chains outer aqueous phase. Their major components are usually phospholipids, with or without cholesterol, mimicking the physiological composition of biomembranes [66]. The most noteworthy advantages of liposomes are represented by their biocompatibility and safety due to their resemblance to biomembranes. The organized structure of liposomes offers the ability to load and deliver molecules with different solubility, with hydrophilic molecules placed into the aqueous core, hydrophobic molecules into the lipid layers, and amphiphilic molecules at the water/lipid bilayer interface [67]. According to the structure of the lipid bilayers of the vesicles, liposomes are commonly classified into unilamellar (ULV, all size range), multilamellar (MLV, >500 nm), and multivesicular (MVV, >1000 nm) vesicles (Figure 4).

Based on vesicles size, ULV can be furtherly divided into three classes named small unilamellar vesicles (SUV, 20–100 nm), large unilamellar vesicles (LUV, >100 nm), and giant unilamellar vesicles (GUV, >1000 nm) (Figure 4). ULV is characterized by the presence of a single phospholipid bilayer that can encapsulate hydrophilic molecules, while MLV presents two or more concentric lipid bilayers characterized by an onion-like structure that can hold lipophilic compounds [68]. It is possible to modify the liposomal surfaces by conjugation to polymers and/or ligands to provide special properties such as active targeting to specific sites. 

Liposomes whose size does not exceed the scale of 100 nm are called nanoliposomes. Elastic liposomes (EL) are vesicles characterized by flexibility, deformability, or ultradeformability. ELs were introduced in 1992 by Cevc and Blume as an alternative to conventional liposomes to facilitate drug passage across the stratum corneum of the skin [69].

Compared to conventional liposomes, it was found that ELs exert greater biomembrane crossing capabilities, including BBB, due to their small size and elastic nature [70].

ELs were first developed as novel and transdermal DDS; indeed, their elasticity enables them to cross membrane pores smaller than their own size by incorporating edge activators (surfactants) into lipid bilayers. Sodium cholate, Span 80, or Tween 80 were employed as surfactants [70]. The enhanced permeability of EL is due to their ability to act as carriers and penetrant agents. Surprisingly, ELs can penetrate the skin without disintegration [71].

During the last decades, different types of molecules (i.e., thermolabile proteins, acid-labile drugs, enzyme-susceptible, highly lipophilic, hydrophilic, photosensitive drugs, and high-molecular-weight molecules) have been encapsulated within EL for different applications [72,73,74]. Altamimi et al. designed EL for luteolin transdermal delivery for breast cancer therapy, demonstrating that the deformable vesicular carrier enhanced permeation parameters across rat skin, exhibiting a concentration-dependent MCF-7 cells inhibition and improving cellular internalization as compared with pure drug solution [75]. Montanari et al. [76] exploited ultradeformable liposomes activated by sunlight for the treatment of *Leishmania braziliensis* infections, demonstrating that the transcutaneous penetration of zinc phthalocyanine was about 10 times higher when encapsulated in ultra-deformable liposomes compared to conventional liposomes, having a homogeneous distribution throughout the stratum corneum [77].

Niosomes are surfactant-based nanometric vesicles with advantageous characteristics compared to conventional liposomes. Niosomes are formed by non-ionic surfactants via self-assembly in an aqueous solution [78]. The use of non-ionic surfactants as membrane-forming constituents instead of phospholipids overcomes many of the disadvantages associated with liposomes, such as chemical instability, predisposition of phospholipids to oxidation, necessity of special handling, and storage conditions. Furthermore, their specific structure, composed of an inner aqueous compartment surrounded by a hydrophobic membrane, allows the incorporation and codelivery of hydrophobic and hydrophilic molecules. However, niosomes show some disadvantages, such as greater irritability compared to liposomes and lower biocompatibility than phospholipids due to the presence of surfactants [79]. Niosomes first emerged in the field of cosmetics by researchers from L’Oréal (Clichy, France) in the 1970s and 1980s. Since then, niosomes have been extensively investigated and are now attracting extensive attention as a vesicle delivery system for multiple applications in different fields, including pharmaceutical, cosmetic, and food sciences, leading to a large number of publications and patents [80,81,82].

Phytosomes, also called phyto-phospholipid complexes, are vesicular systems formed by the interaction between the hydrophilic parts of phospholipids and the phyto-active components, resulting in the formation of hydrogen bonds between them. The hydroxyl groups of polyphenols, or phytochemicals, produced by plants form hydrogen bonds with nitrate and phosphate groups of phospholipids. Phytosomes have a different structure compared to liposomes since the active ingredient is not located inside the hydrophilic cavity or within the layers of membranes, like in liposomes, but it is part of the membrane itself [83]. The chemical bonding ensures the stability of phytosomes and enhances the encapsulation efficiency of the bioactive compounds, generally at a stoichiometric molar ratio of 1:1 or 1:2 (phospholipids/phytochemicals) [84].

The lipid bilayer of the phytosomes helps contact-facilitated drug delivery in which there is a lipid–lipid interaction between the carrier and the cell membrane, leading to the diffusion of the bioactive compounds into the cell. The rate of release is slower than liposomes due to the association of the drug with the phosphatidyl head [85].

The first phytosomes were developed by Indena company (Milan, Italy) in the late 1980s, which aimed to increase the bioavailability of phytochemicals by complexing them to phospholipids [86]. Different phytosomes are available in the market (sylibin, ginkgo, cartaegus, and centella), and many others are currently under investigation in clinical trials, as detailed in a very interesting recently published review by Alharbi et al. [86].

The biological activities related to phytosomes are heterogeneous and involve different districts such as cardiovascular, central and peripheral nervous, gastrointestinal, genitourinary, immune, integumentary, musculoskeletal, and respiratory systems. For example, Panda and Naik investigated the cardioprotective activity of a combined treatment of *Ginkgo biloba* phytosomes and *Ocimum sanctum* extract in isoproterenol-induced myocardial necrosis in rats, showing an evident cardioprotective effect [87].

Polymerosomes are artificial vesicles enclosing an aqueous cavity formed by the self-assembly of amphiphilic natural or synthetic copolymers. Polymers are chemical compounds consisting of many repeating subunits called monomers and exist as chains or in branched form. Block copolymers are macromolecules that contain different adjacent blocks of chemically distinct monomers. Block copolymers contain both hydrophilic and hydrophobic blocks that possess amphiphilic properties [88]. Polymerosomes can have a size in the order of nanometric or micrometric scale, depending on the preparation method used, the amphiphilicity of polymers themselves, or external factors such as extrusion or sonication process during their synthesis. Polymerosomes have higher stability than liposomes and can be used to obtain more controlled release kinetics, also in a stimuli-response manner. The release of the drug can be triggered by several different factors, such as pH, temperature, redox potential, light, magnetic field, or instability of the system [89]. Polymerosomes can be loaded with hydrophilic, hydrophobic, or amphiphilic compounds, which makes them very attractive vesicles for various applications in drug delivery.

Use of polymersomes for drug delivery and targeting requires several steps that consists in the synthesis of amphiphilic block-copolymers, assembly of block-copolymers to form vesicles, and in some cases can include targeting of the vesicles by conjugation of specifically binding moieties, and strategies for controlling the release of drugs from polymersomes by the use of internal or external stimuli (hyperthermia and magnetic field-induced release, ultrasound-induced release, light-induced release, voltage-induced release) [90].

Polymersomes can also be designed for other purposes. These include the encapsulation of diagnostic markers, enzymes, or other reactive molecules (nanoreactors and artificial cells). Liu et al. investigated the use of polymersomes as a targeted contrasting agent for magnetic resonance imaging (MRI). The authors prepared folate-tagged poly(L-glutamic acid)-block-poly(ε-caprolactone) vesicles and subsequently formed superparamagnetic iron oxide (Fe_3_O_4_) NPs in their hydrophilic crowns. These superparamagnetic polymersomes successfully contrasted transplanted HeLa tumors in mice [91].

### 3.2. Metallic Nanoparticles

**Figure 5:**
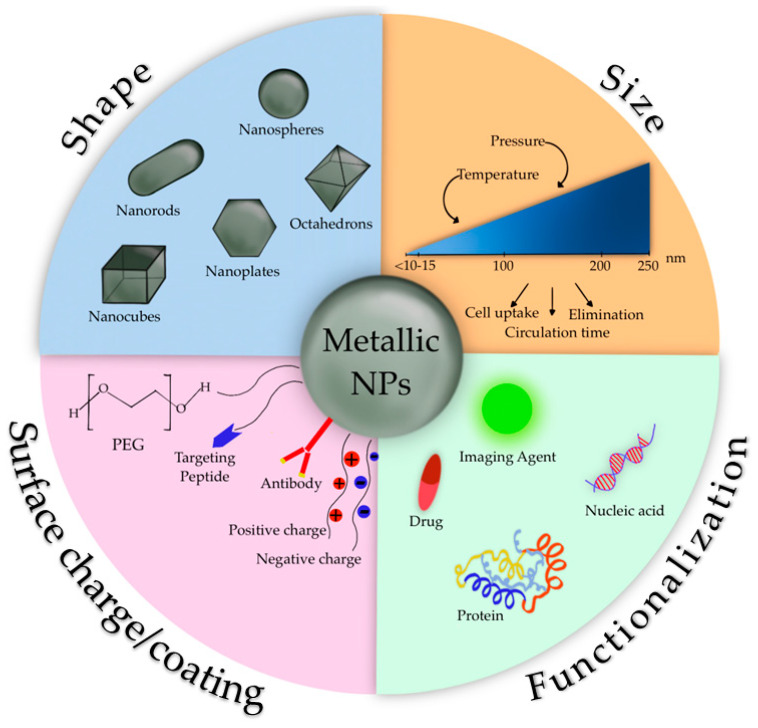
Characteristics of metallic NPs.

Metallic NPs represent an emerging category of DDS with a wide range of potential applications in biotechnology, targeted drug delivery, gene delivery, and diagnostic imaging. In 1857, Faraday first investigated the existence of metallic NPs in solution [92]. In 1908, Mie gave a quantitative explanation of their color [93]. Nowadays, these nanosystems can be prepared and modified with various chemical functional groups, which allows them to bind with antibodies, ligands, and/or drugs (Figure 5).

These systems can be made of pure metals (e.g., gold, platinum, silver, titanium, zinc, cerium, iron, and thallium) or their compounds (e.g., oxides, hydroxides, sulfides, phosphates, fluorides, and chlorides) [94].

During the last five decades, magnetic Fe_3_O_4_ NPs (MNPs) have been highly investigated mainly due to their optical, thermal, and magnetic properties, along with the fact that they can be manipulated with an external magnetic field. Among eight forms of Fe_3_O_4_, magnetite NPs are one of the most representative MNPs, having unique catalytic, biological, and magnetic properties such as the superparamagnetic ones. Superparamagnetic NPs only exhibit size-dependent magnetic features when exposed to an external magnetic field, while bulk magnetic particulates retain these features even without an external magnetic field [94].

The physico-chemical features of MNPs strongly depend on their size and shape; these features, along with coating molecules type and surface charge, influence pharmacokinetics and pharmacodynamics after in vivo administration [95]. In order to avoid the elimination by the mononuclear phagocytic system after MNPs administration [95] and to enhance their stability in vivo, different coating agents have been considered. The surface coating of MNPs drives their intracellular trafficking and degradation in endolysosomes, as well as dictating other cellular outcomes. Coating molecules into the MNPs surface can be useful to avoid their opsonization and to reduce their aggregation and agglomeration that impair the interaction with the cellular compartment. Several coating agents have been evaluated, for example, inorganic compounds, such as silica, that can enhance MNPs biocompatibility and stability, while metal conjugation of Fe_3_O_4_ with gold gives multifunctionality.

Organic compounds such as polyethylene glycol (PEG) and derivatives prevent plasma proteins adsorption onto MNPs surface, avoiding their uptake; other synthetic polymers like poly (D,L-lactide-co-glycolic) acid-based (PLGA) may also be used for other purposes. Polymer-functionalized MNPs have improved stability due to the increased repulsion, which provides an equilibrium of the magnetic and attractive forces. The functional groups of coating compounds, such as hydroxyl, carboxylic, or amine groups, offer MNPs the possibility to bind drugs, proteins, or biomolecules, providing reactive sites on the surface of MNPs potentially useful for the attachment of therapeutic agents [95].

Among metallic NPs, gold NPs (AuNPs) have piqued great interest due to their many advantages, such as the simplicity of synthesizing NPs with different shapes (i.e., rod-like, spherical, and cage-like) and tunable size, which confers optical and electrical properties. Additional characteristics of AuNPs are the net negative surface charge that allows the functionalization with biomolecules such as targeting ligands, the biocompatibility, and their surface effect, including macroscopic quantum tunneling effect and the presence of surface plasmon resonance bands [96]. Recent studies have shown that AuNPs not only can infiltrate the blood vessels but also enter inside the organelles, suggesting they can be employed as effective drug carriers. Small molecules, peptides, oligonucleotides, and DNA can be conjugated with AuNPs, obtaining an efficient release of these payloads via internal or external stimuli [97]. It has also been reported that encapsulating drugs or peptides into AuNPs can improve their bioavailability and biocompatibility [98].

AuNPs are increasingly actively employed for therapeutic reasons. Accumulation of AuNPs in the tumor is highlighted by the modification in the color of the tumor, which appears as a bright red color (typical of colloidal gold and its aggregates) [99]. Interestingly, it has been reported that AuNPs have favorable impacts on plant growth and development and have been recommended for use in a variety of agricultural crops, as well as in the germination of seeds from endangered plant species [100]. Arora et al. demonstrated that spraying AuNPs at concentrations of 10 and 25 mg/L on *Brassica juncea* plants can increase the quantity of chlorophyll and present a viable alternative to genetically modified crops for ensuring food security [101]. 

### 3.3. Derivative Conjugates

Compared to the majority of small molecules, peptides demonstrate short blood half-life due to their susceptibility to enzyme cleavage and rapid renal clearance [101]. Several strategies have been widely used to improve the chemical and physical stability of peptides and their pharmacokinetics and pharmacodynamics.

One of the most effective ways to prevent the degradation is to engineer analogs from dextrorotatory (D)-amino acids; in fact, these latter show improved stability among proteases [102]. However, it is important to consider the effects that such modifications could have on the overall secondary structure of the peptide, which risks losing the correct binding geometry to its target. 

Backbone modifications [103], cyclization [104], lipidation [105], introduction of differently sized polymers [106], and conjugation represent additional strategies employed to improve peptide properties.

Conjugation of peptides with other peptides, small molecules, or biomolecules represents an essential tool in biomedical research. It can be used to promote cellular uptake or receptor-mediated drug delivery and/or to extend the peptide half-life in the bloodstream.

It has been reported that attaching moieties capable of increasing the size of peptides and/or altering their charge has the potential to successfully extend its blood half-life. Conjugates used in peptide therapeutics can either be non-biological molecules such as PEG or biological molecules such as sugars, proteins, or lipids. Increasing the molecular weight of peptides allows them to evade kidney filtration; otherwise, attaching a conjugate that is negatively charged helps to avoid renal clearance [107]. Conjugates that stabilize the structure of the therapeutic peptide can help to escape enzymatic degradation. 

Conjugation is useful to facilitate site-specific transport to different tissues. It has been shown that animal lectins and galectins are important mediators in inflammatory diseases in recognition processes; it has prompted to synthesize glycoconjugates of small molecules or peptides, such as carnosine, with small sugars including glucose, lactose, or trehalose, to be specifically bound to a selected lectin [108].

Peptide-drug conjugates (PDCs) are a class of targeted therapeutics for cancer treatment, which can also be used as successful diagnostic tools in various scanning techniques by including radionuclides in their structure. PDCs consist of a homing peptide, which is chosen depending on its specific targeting properties, a cleavable or non-cleavable linker, and a cytotoxic payload [109].

Conjugation of a peptide with drugs can be further used to improve the effectiveness and reduce the side effects of the drug. Kulikova et al. formulated a synthetic derivative of acetylsalicylic acid and carnosine to take advantage of the superoxide scavenging and antiplatelet activities of carnosine to limit the adverse effects of the acetylsalicylic acid in the gastrointestinal tract [110].

**Figure 6:**
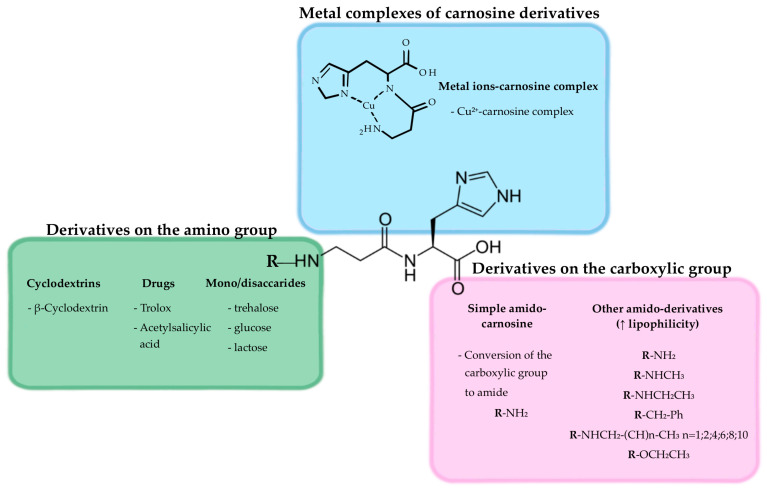
Graphical representation of potential carnosine derivatives.

In Figure 6, examples of other carnosine derivatives were depicted, while their detailed synthesis and applications can be found in the next section.

## 4. Increasing Carnosine Bioavailability through DDS and/or Chemical Modifications

As previously mentioned, the therapeutic potential of carnosine is reduced as a consequence of its low stability/bioavailability due to CNDP1 and CNDP2 activity, metabolizing the dipeptide into its two constituting amino acids at sieric and intracellular levels, respectively. Because of that, different researchers have been working on the development of new approaches or new formulations trying to improve carnosine bioavailability as well as target selectivity (via DDS). One of the approaches considered was the use of selective inhibitors of carnosinases, as in the case of carnostatine (SAN9812) [111]. This protease-directed small-molecule was able to inhibit CNDP1 activity in human serum as well as in serum obtained from transgenic mice-overexpressing human CNDP1. In the same study, the authors were able to demonstrate that the simultaneous administration of carnosine and SAN9812 significantly increased the levels of carnosine in both plasma and kidney (up to 100-fold vs. treatment-naïve CNDP1-overexpressing mice). The ability of reduced glutathione (GSH), N-acetylcysteine, and cysteine to inhibit CNDP1 activity has also been considered. As shown by Peters and collaborators, these molecules have a dose-dependent effect in decreasing the efficiency of a recombinant CNDP1, also normalizing the increased activity of this peptidase in renal tissue samples obtained from diabetic mice [112]. Further investigations allowed to demonstrate that the inhibition of CNDP1 was allosteric.

As recently described by Grasso et al. [19], alternative and innovative approaches aiming at increasing carnosine bioavailability and/or its delivery consider the use of carnosine derivatives, vesicular systems, or nanoparticulate systems (Table 2), all described in detail in the next sub-sections.

### 4.1. Vesicular Systems

Different vesicular systems vehiculating carnosine, including nanoliposomes, liposomes, niosomes, proniosomes, polymerosomes, phytosomes, and nanophytosomes, have been investigated (Table 2).

Maherani et al. studied how lipid composition can influence the physico-chemical properties of nanoliposomes encapsulating carnosine [113]. In order to increase the encapsulation efficiency of carnosine, nanoliposomes were prepared considering the effects of 1,2-dioleoyl-sn-glycero-3-phosphocholine (DOPC), 1,2-dipalmitoyl-sn-glycero-3-phosphocholine (DPPC), and 1-palmitoyl-2-oleoyl-sn-glycero-3-phosphocholine (POPC) on vesicles’ size, zeta potential, phase transition temperature, and fluidity, with DOPC and DPPC providing the best results in terms of size and encapsulation efficiency.

Anti-inflammatory and antioxidant effects of liposomal and non-liposomal carnosine in adjuvant arthritis were compared by Slovák et al. [114]. Both forms were able to decrease plasmatic levels of interleukin (IL)-1β, matrix metalloproteinase-9, and monocyte chemoattractant protein-1 (MCP-1), but only liposomal carnosine significantly reduced the levels of MCP-1. Of note, liposomal carnosine was more effective in counteracting oxidative stress in plasma as well as in decreasing the mRNA expression of inducible NO synthase in cartilage tissue compared to free carnosine. Neuroprotective effects of carnosine-loaded EL, prepared by extrusion method using egg phosphatidylcholine (phospholipid) and Tween 80 (edge activator), have instead been shown in a cerebral ischemia rat model [115]. Carnosine-loaded EL, having elasticity two-fold higher compared to conventional liposomes, was characterized by nanometric particle size close to 100 nm and homogeneous distribution, also showing a polydispersity index below 0.1. The elasticity of CAR-ELs was 2-fold higher than that of conventional liposomes.

A lipoic acid-based transient receptor potential ankyrin type-1 antagonist, obtained by condensing carnosine with lipoic acid, has been encapsulated into niosomes for brain targeting [25]. In a different study, carnosine and carnosine-loaded niosomes were investigated to evaluate their activities, including the ability to inhibit bovine serum albumin (BSA) aggregation [116]. Carnosine-loaded niosomes were demonstrated to be an efficient drug delivery platform for simultaneous BBB crossing along with the ability to reduce oxidative stress, measured in terms of advanced glycation end-products (AGEs) and advanced oxidation protein products formation, inflammation, and BSA aggregation [25,116,117].

Kim et al. have encapsulated carnosine in lipoprotein receptor-related protein-1 (LRP-1)-targeted functionalized polymersomes and investigated their effects in an in vivo ischemic stroke rat model [118]. Carnosine-loaded polymersomes reduced the aggregation of LRP-1 at the brain level and exhibited remarkable neuroprotective effects despite a dose of carnosine three orders of magnitude lower than the free form.

A study published in 2016 describes carnosine-loaded phytosomes as an alternative to the prodrug N-acetyl-carnosine as a novel delivery system to the lens [119]. It is worth mentioning that, as observed by analyzing ex vivo transcorneal permeation parameters, carnosine-loaded phytosomes showed significantly controlled corneal permeation without changes in primary human corneal cell viability. The same study showed the ability of these formulations to inhibit the brunescence of porcine lenses incubated in a high-glucose medium, indicating the potential for delaying changes that underlie cataractogenesis.

Recently, Darvishi and collaborators demonstrated the synergic effect of dual delivery of carnosine and aloe vera into nanophytosomes in enhancing the protective activity against methylglyoxal-induced angiogenesis impairment in human umbilical vein endothelial cells (HUVECs) [120]. Carnosine/aloe vera-loaded nanophytosomes decreased the toxicity induced by methylglyoxal in HUVEC cells and showed improved free radical scavenging potency and NO synthesizing capacity; these effects were paralleled by enhanced proangiogenic activity as showed by the increased expression of hypoxia-inducible factor 1-alpha (HIF-1α), vascular endothelial growth factor A (VEGFA), basic fibroblast growth factor (bFGF), kinase insert domain receptor (KDR), and Angiotensin II (Ang II) genes.

### 4.2. Nanoparticles

The use of MNPs coated with carnosine has been considered to obtain both a higher stability of the colloidal suspension and an enhanced therapeutic effect of carnosine [121] (Table 3).

Carnosine-coated Fe_3_O_4_ NPs, obtained through the co-precipitation of Fe_3_O_4_ in the presence of the dipeptide, have been considered for different applications, including cell separation, diagnosis, and targeted drug delivery for cancer therapy [122]. In a different study, carnosine functionalized Fe_3_O_4_ NPs loaded with dexamethasone were studied as a possible drug delivery platform for simultaneous BBB crossing [123]. The authors investigated the possible cytotoxic effects to obtain information regarding their biocompatibility in drug delivery in the context of brain damage. The efficacy of BBB carriers was demonstrated and the drug release study for ischemic stroke treatment was presented.

Stimuli-responsive MNPs coated with carnosine have been synthetized and tested, both in vitro and in vivo, for breast cancer therapy [121]. The new formulation was characterized by colloidal stability and the absence of agglomeration issues. When tested in vitro on human breast cancer cells, carnosine-coated MNPs displayed a higher cytotoxic activity compared to free carnosine. Promising results were also obtained in vivo, where carnosine-coated MNPs were able to significantly reduce the size of the tumor without inducing systemic toxicity. The authors were also able to demonstrate an enhanced anti-angiogenic activity of the new formulation. Recently, Khramtsov et al. carried out a research study in which they synthesized nanoclusters of magnetic iron-carbon NPs coated with different proteins, analyzed their physico-chemical properties, and used nanoclusters conjugated with recombinant protein G from *Streptococcus* sp. as labels in a nuclear magnetic resonance immunoassay of IgG against the tetanus vaccine [124]. All four protein coatings (BSA, casein, and gelatins A and B) provided the nanoclusters with long-term storage stability, paralleled by good stability in physiological media and high relaxivity.

A new formulation of carnosine with biotin (BioCar), resistant to the degrading activity of carnosinases in human plasma, was made and structurally characterized by Bellia et al., with the main aim to take advantage of the avidin-biotin technology allowing for the selective delivery of biotinylated agents [125]. In this study, the binding affinity to avidin and streptavidin was used for the functionalization of avidin- and streptavidin- AuNPs with BioCar.

The synthesis, characterization, and cytotoxic effects of AuNPs and their loading with N-acetyl-carnosine for the treatment of cataract has also been performed [126]. The encapsulation of N-acetyl-carnosine into AuNPs significantly increased both biocompatibility and bioavailability without toxic effects when tested on fibroblast cells.

The ability of carnosine to inhibit the proliferation of glioblastoma U87 cancer cells and then to reduce the risk of metastasis has been recently demonstrated by Habra et al. [128]. The same authors have also investigated the controlled release of carnosine from poly(lactic-co-glycolic acid) beads using nanomechanical magnetic triggers [127]. The possibility of obtaining a safe and triggered release of onsite drug delivery of these new drug-delivery vesicles as part of a theragnostic treatment for glioblastoma was also proposed.

PEGylated liquisomes have been proposed as a novel combined passive targeting nanoplatform of carnosine for breast cancer [129]. These formulations were able to protect carnosine from degradation in vivo, prolonging its release and enhancing its anti-cancer activity (% tumor growth, VEGF, cyclin D1, and caspase-3 tissue levels) compared to free carnosine.

### 4.3. Derivatives/Conjugates

During the last two decades, different derivatization strategies aiming at increasing carnosine activity and its stability to carnosinases (e.g., derivatized with sulfamido pseudopeptides [130]), representing an important limit for the therapeutic use of this molecule [17], have been considered (Table 4).

For example, the use of cyclodextrins (CDs) to synthesize carnosine derivatives has been exploited to enhance carnosine activity, with the ability of CDs to scavenge hydroxyl radicals being synergic with the antioxidant activity of carnosine [132]. The amino group of the β-alanine or the carboxyl group of the histidine can be both derivatized to obtain therapeutic carnosinase-resistant molecules. The addition of conjugates can confer a steric shielding effect against proteases and peptidases.

Several carnosine derivatives with saccharides, such as β-CD and trehalose, have been synthesized [108] (a graphic representation of β-CD-carnosine conjugate is depicted in Figure 3).

Different carnosine derivatives with β-CD have been synthesized and structurally characterized, but only a few of them have been tested in biological systems. More than 20 years ago, Vecchio et al. described the synthesis and conformation of β-CDs functionalized with enantiomers of Boc-carnosine [131], suggesting that the CD could represent a stabilizer and a carrier of the dipeptide. The authors further suggested that the moiety of carnosine, as well as the Boc group, may make β-CD-carnosine derivatives far more efficient artificial chaperones compared to free CDs because of the occurring hydrogen bond interactions. Numerous preclinical studies have shown that carnosine and its analog homocarnosine (beta-aminobutyril-L-histidine) are able to scavenge reactive oxygen species. In a study by Amorini et al., the synthesis and antioxidant activity of new homocarnosine β-CD conjugates were described [132]. β-CD-carnosine derivatives demonstrated a higher ability to inhibit the Cu^2+^-driven low-density lipoprotein (LDL) oxidation compared to homocarnosine derivatives. An additional study related to this topic considered the synthesis of β-CD-carnosine derivatives and their hydroxyl radical scavenger ability [133]. By using pulse-radiolysis, it was shown that the new derivatives of carnosine considered are excellent scavengers of hydroxyl radical, with the activity coming from both the glucose moieties of the macrocycle and the formation of the stable imidazole-centered radical. The ability of β-CD-carnosine derivatives to interact with Cu^2+^ as well as that of this transition metal ion to induce the formation of supramolecular assemblies (β-CD-carnosine oligomeric species up to hexamer) have also been demonstrated [134]. Despite these promising results obtained by using cell-free systems, it remains to elucidate their therapeutic properties in biological systems such as cells in the absence or in the presence of pro-oxidant stimuli.

With regards to trehalose–carnosine conjugates, recently, the ionophore ability of a trehalose conjugate to activate tyrosine kinase cascade pathways and assist copper signal in triggering brain-derived neurotrophic factor (BDNF) and VEGF activation in PC12 cells has been proved [145]. In a different study, trehalose–carnosine conjugates ability to inhibit amyloid-β (Aβ) aggregation, tune Cu^2+^ activity, also decreasing the toxic effects exerted by acrolein has been described [144]. The newly synthesized glyco-conjugate (TrCar2) was resistant to human carnosinase hydrolysis, quenched acrolein and their Cu^2+^ complexes, showed superoxide dismutase (SOD)-like activity, and inhibited both self- and metal-induced Aβ aggregation. As for the latter activity, it has also been demonstrated for hyaluronan-carnosine conjugates [146]. These derivatives showed an inhibitory activity higher than the parent compounds, with an effect proportional to the loading of carnosine. Of note, hyaluronan–carnosine conjugates were also able to dissolve the amyloid fibrils and reduce Aβ-induced toxicity in undifferentiated SH-SY5Y cells. Numerous studies have been devoted to the investigation of N-acetyl-carnosine activity and, in particular, its ability to counteract oxidative stress [135,136,137]. This ability was also paralleled by decreased DNA damage [135], decreased lipid peroxidation [136], or reduced glycation process and AGEs formation [137]. The inhibition of AGEs has been proved in vivo by FL-926-16, a novel bioavailable carnosinase-resistant carnosine derivative [150]. This derivative also demonstrated the ability to prevent the onset and to block the progression of diabetic nephropathy in *db*/*db* mice by quenching reactive carbonyl species, thus reducing the accumulation of their protein adducts and the related inflammatory response (including the NLRP3 inflammasome).

Additional derivatives of carnosine able to significantly counteract oxidative stress phenomena are represented by carnosine derivatized with Trolox [142], vitamin E (VECAR) [143], or acetylsalicylic acid [110] as well as lipoilcarnosine [141], carnosine analogs containing NO-donor substructures [149], and amide derivatives [148]. The latter derivatives have also been shown to be able to regulate metal homeostasis [108]. Another study carried out by Anderson et al. described the pharmacological effects of carnosinol, a derivative of carnosine characterized by high oral bioavailability, in a model of diet-induced obesity and metabolic syndrome [147]. Carnosinol decreased the formation of 4-hydroxynonenal (HNE) adducts in both liver and skeletal muscle in a concentration-dependent manner, also counteracting other alterations, including inflammation and insulin resistance.

Improved self-assembling and complexing processes, along with increased amphiphilic hydrogelation, have been described for histidine-based derivatives inspired by carnosine [140]. Improved self-assembling was also demonstrated for carnosine derivatized by acylation with palmitoyl chain [138], while enhanced amphiphilic hydrogelation, including the ability to efficiently gelate water, was observed in the case of carnosine derivatized by acylation with benzoic acid [139].

## 5. Conclusions and Future Perspectives

Drug development is currently focused on the identification of novel carnosine formulations able to improve its efficacy and stability, finally increasing its therapeutic potential in humans. Nowadays, the use of innovative DDS and/or the chemical modifications of carnosine led to the development of formulations allowing to decrease, or in the best scenario, completely prevent its hydrolysis by carnosinases, the simultaneous BBB crossing, to maintain or enhance carnosine biological activity, as well as the site-specific transport to different tissues. Despite these promising results, further studies carried out both in cells challenged with specific pro-oxidant/pro-inflammatory stimuli and in animal models of systemic and neurodegenerative disorders are needed to translate these findings to clinical practice.

## Figures and Tables

**Table 1:** Main advantages and limitations of vesicular systems, metallic NPs, and drug–conjugate derivatives.

		Advantages	Limitations
**Vesicular Systems**	**Liposome**	Made of natural ingredients; Biodegradable and biocompatible; Similarity to biomembrane.	Physical instability during storage; Susceptible to oxidation; Rigid liposomes remain confined to the stratum corneum.
	**Elastic ** **Liposome**	Highly deforming ability and flexibility ensure deeper skin penetration and biomembrane crossing ability.	On prolonged storage, due to increased elasticity and flexibility, it tends to be less stable and lose the entrapped drug, which complicates the scaling process.
	**Niosome**	Chemical stability.	Lower biocompatibility.
	**Phytosome**	Enhancement of pharmacokinetic and pharmacodynamic properties of herbal-originated polyphenolic compounds; Improves skin absorption of phytoconstituents;	Despite the easy scale-up production of phytosomes, the high pH sensitivity of some components could limit the large-scale synthesis of such formulations and should be considered during the manufacturing.
		Better stability of incorporated compounds owing to the chemical interaction.	
	**Polymerosome**	More stable than liposomes; Allows greater control of chemical and structural properties; Can be used to obtain controlled release kinetics by stimuli-response triggers.	In case of charged polymers, the self-assembled polymersome could induce stronger immune response and therefore be less tolerable for medical applications.
**Metallic NPs**		Multiple shapes; Conductivity; Localized surface plasmon resonance; Ability to direct uptake through external magnetic stimulation.	Chemical contaminants from synthesis can cause toxicity issues.
**Drug-conjugate derivatives**		Increased compound half-life; Increased target specificity; Increase drug stability.	Modification can reduce the potency, especially for small peptides and proteins; Any covalent modification of peptides or proteins presents a potential risk of increased immunogenicity.

**Table 2:** New vesicular system-based formulations of carnosine.

Formulation Name	Basic Description	Mode of Action	Ref.
Nanoliposomes	Carnosine incorporated into nanoliposomes could represent an innovative approach to overcoming the issues related to the direct application of this antioxidant peptide in food.	▪ Increased encapsulation efficiency	[113]
Liposomes	Liposomes are nanosized vesicles with a spherical shape that can be produced starting from natural or synthetic phospholipids. Encapsulation of antioxidants into liposomes has been shown to improve their therapeutic potential against oxidant-induced tissue injuries, facilitating intracellular delivery and extending the retention time of incorporated agents inside the cell.	▪ Reduced oxidative stress and inflammation	[114]
Elastic liposomes	EL encapsulated with carnosine represent a promising strategy to enhance the transport into the brain, protecting the dipeptide against enzymatic hydrolysis.	▪ Exerted neuroprotection	[115]
Niosome derivatized with lipoyl-carnosine	Niosomes are nanovesicles coupled to specific ligands selectively recognized by transporters expressed on the BBB that could promote the delivery of drugs (e.g., carnosine) at brain level.	▪ Drug delivery for simultaneous BBB crossing ▪ Reduced oxidative stress	[25]
Niosome	Carnosine-encapsulated niosomes represent a powerful drug delivery tool allowing it to reach specific organs such as the brain.	▪ Decreased oxidative stress and inflammation (AGEs and AOPP) ▪ Anti-aggregation (BSA)	[116,117]
Proniosome	Proniosomes are non-hydrated niosomes, which, upon hydration, form niosomes characterized by physical stability that overcome some problems presented by other vesicular systems, such as leaking, fusion, and aggregation.	▪ Increased bioavailability	[117]
Polymerosome	Polymersomes are synthetic vesicles formed through the self-assembly of amphiphilic co-polymers in aqueous conditions. Carnosine encapsulated in polymersomes could exert an enhanced neuroprotective potential.	▪ Exhibited remarkable neuroprotective effects with a dose of carnosine 3 orders of magnitude lower than free carnosine ▪ Reduced aggregation in the brain (LRP-1 target)	[118]
Phytosome	Carnosine loaded into lipid-based phytosomes represent an alternative for the prodrug N-acetyl-carnosine as a novel delivery system to the lens.	▪ Increased corneal permeation	[119]
Nanophytosome	Nanophytosomes represent one of the novel nanocarriers that could provide potent applications in both food and pharmaceutical fields. A novel nanophytosomal formulation obtained by physical mixture of two compounds, carnosine and Aloe vera, has shown a synergic effect in counteracting cell toxicity.	▪ Decreased oxidative stress and toxicity ▪ Increased proangiogenic activity (i.e., HIF-1α, VEGFA, bFGF, KDR, and Ang II genes)	[120]

**Table 3:** New metallic nanoparticle-based formulations of carnosine.

Formulation Name	Basic Description	Mode of Action	Ref.
Fe_3_O_4_	Carnosine-coated Fe_3_O_4_ NPs have been prepared via co-precipitation of Fe_3_O_4_ in the presence of carnosine. They are commonly used because of their superparamagnetic properties allowing potential applications in many fields.	▪ Enhanced *ac* conductivity	[122]
Fe_3_O_4_ NPs/poly(lactic-co-glycolic acid) (PLGA) polymer-loaded dexamethasone functionalized with carnosine	PLGA functionalized Fe_3_O_4_ NPs with carnosine peptide composite loaded with dexamethasone represent suitable drug delivery carriers for biomedical applications able to improve the therapeutic efficiency of carnosine.	▪ Drug delivery for simultaneous BBB crossing	[123]
Magnetic	Carnosine-coated MNPs were developed to enhance the chemotherapeutic activity of this dipeptide.	▪ Enhanced toxicity	[121]
		▪ Improved physicochemical properties	[124]
AuNPs/biotin	A new carnosine derivative with biotin was synthesized and structurally characterized. The binding affinity of the new molecular entity to avidin and streptavidin was exploited to functionalize avidin- and streptavidin-AuNPs with the carnosine–biotin conjugate.	▪ Chelating activity (Cu^2+^ and Zn^2+^) ▪ Biotin-like affinity for avidin and streptavidin	[125]
AuNPs/N-acetyl-carnosine	NPs loaded with N-acetyl-carnosine were synthesized, characterized, and tested for cataract treatment. The AuNPs were biofabricated and characterized by using *Coccinia grandis* bark extract.	▪ Increased biocompatibility and bioavailability of drug ▪ Reduced toxicity	[126]
Poly (lactic-co-glycolic acid) microbeads	The Fe_3_O_4_ NPs have been encapsulated, along with carnosine, inside porous poly(lactic-co-glycolic acid) microbeads. These new drug-delivery vesicles have the potential to pave the way towards the safe and triggered release of onsite drug delivery as part of a theragnostic treatment for cancer.	▪ Increased bioavailability of drug ▪ Enhanced intracellular uptake	[127]

**Table 4:** New carnosine formulations obtained by derivatization/conjugation.

Formulation Name	Basic Description	Mode of Action	Ref.
Derivatized with β-cyclodextrins	β-cyclodextrin is a heptasaccharide derived from glucose. Ciclodextrins are particularly used in pharmaceutical science for their ability to include and/or stabilize drugs. Glycoconjugate derivatives obtained by functionalization with carnosine in different positions of the sugar or the cyclodextrin are widely used because of their decreased susceptibility to degradation by carnosinases.	▪ Antioxidant activity at concentrations 10–20 times lower than that reported for other synthetic derivatives	[131,132,133]
		▪ Cu^2+^ modulation of carnosine derivative oligomeric species formation	[134]
N-acetylcarnosine	N-acetyl-carnosine is obtained by the addition of an acetyl group to carnosine structure, which makes the dipeptide more resistant to the degradation exerted by carnosinases.	▪ Decreased oxidative stress and DNA damage	[135]
		▪ Reduced oxidative stress and lipid peroxidation ▪ Anti-aging	[136]
		▪ Reduced oxidative stress ▪ Reduced glycation process and AGEs formation	[137]
		▪ Decreased oxidative stress and lipid peroxidation ▪ Inhibition of UVB Erythema	[110]
Derivatized by acylation with palmitoyl chain	Palmitic acid is a fatty acid with a 16-carbon chain. This compound is commonly used as a structure-directing agent to induce the fibrillization of carnosine. Its long lipid chains are able to drive self-assembly due to amphiphilicity, showing restricted dynamics and/or crystallization.	▪ Improved self-assembly into nanotapes	[138]
Derivatized by acylation with benzoic acid	Benzoic acid is a compound comprising a benzene ring core carrying a carboxylic acid substituent. N-(4-n-tetradecyloxybenzoyl)-L-carnosine represents a carnosine-based amphiphilic hydrogelator that efficiently gelates water and exhibits salt, pH, and thermoresponsive gelation properties.	▪ Increased amphiphilic hydrogelation	[139]
Histidine-based derivatives	Novel histidine-based complexing surfactants containing trifunctional moduli (peptidic/hydrophilic/hydrophobic). It is possible to establish various links between the different parts, allowing the modulation of the lipophilic/hydrophilic balance and obtaining amphiphilic compounds with complexing properties and surfactive or gelator properties.	▪ Improved self-assembling and complexing processes ▪ Increased amphiphilic hydrogelation	[140]
Lipoilcarnosine	Lipoic acid is an organosulfur compound derived from caprylic acid. Lipoilcarnosine is a conjugated molecule obtained by coupling α-lipoic acid to carnosine.	▪ Reduced oxidative stress and toxicity	[141]
Derivatized with trolox	Trolox (6-hydroxy-2,5,7,8-tetramethylchroman-2-carboxylic acid) is a water-soluble analog of vitamin E. (S)-trolox-L-carnosine (STC) and (R)-trolox-L-carnosine (RTC) represent novel derivatives of carnosine synthesized by N-acylation of carnosine with (S)- and (R)-trolox, respectively.	▪ Decreased oxidative stress	[142]
Derivatized with vitamin E-carnosine (VECAR)	VECAR is a novel heterodimer of α-tocopherol (vitamin E) and carnosine that was designed by using 13-carbon phytyl-chain to link carnosine to Trolox at the C2 carbon position, maintaining the antioxidant activities of the two components.	▪ Reduced oxidative stress	[143]
Derivatized with acetylsalicylic acid	Acetylsalicylic acid is a nonsteroidal anti-inflammatory drug (NSAID) used to reduce pain, fever, and/or inflammation and as an antithrombotic. Salicyl-carnosine was synthesized by condensation of acetylsalicylic acid and carnosine. Its properties are particularly promising for the potential development of new anti-inflammatory and antithrombotic drugs.	▪ Reduced oxidative stress ▪ Antiplatelet activity ▪ Protected the gastric mucosa against the formation of ulcerative stomach lesions (anti-ulcer activity)	[110]
Derivatized with trehalose	Trehalose is a sugar consisting of two molecules of glucose. The glyco-conjugate trehalose-carnosine (TrCar), differently from carnosine, is not hydrolyzed by human carnosinases. Particular attention has been paid to the characterization of the Cu^2+^ binding features of TrCar.	▪ Inhibited Aβ aggregation and glycation process ▪ Decreased oxidative stress and toxicity ▪ Modulation of Cu^2+^ activity	[144]
		▪ Activated tyrosine kinase cascade pathways ▪ Induced the expression of BDNF and VEGF	[145]
Derivatized with hyaluronic acid	Hyaluronic acid is a linear glycosaminoglycan, an anionic, gel-like polymer, found in the extracellular matrix of epithelial and connective tissues. A derivative obtained from hyaluronic acid and carnosine was considered a pharmacological approach to cure and/or prevent the onset of neurodegenerative disorders.	▪ Inhibited aggregation of Aβ42 and toxicity	[146]
Carnosinol	A derivative of carnosine with high oral bioavailability because of its resistance to carnosinases. Carnosinol displayed a suitable ADMET (absorption, distribution, metabolism, excretion, and toxicity) profile and the greatest potency and selectivity toward α,β-unsaturated aldehydes.	▪ Reduced oxidative stress and metabolic disorders	[147]
Amide derivatives	New family of amide derivatives that are not significantly hydrolyzed by carnosinases. In these derivatives, the sugar moiety can act as a recognition element.	▪ Regulated metal homeostasis	[108]
		▪ Reduced oxidative stress (protect LDL from oxidation catalyzed by Cu^2+^ ion) ▪ Exerted neuroprotection (protect primary mouse hippocampal neurons against HNE-induced death)	[148]
Carnosine analogues containing NO-donor substructures	Carnosine analogs containing NO-donor substructures of which the physico-chemical characterization and preliminary pharmacological profile were carried out. These analogs are characterized by higher resistance to carnosinases’ degradation.	▪ Reduced oxidative stress (protect LDL from oxidation catalyzed by Cu^2+^ ion and HNE scavenging) ▪ Exerted neuroprotection	[149]
Derivatized with sulfamido pseudopeptides	These compounds, characterized by the presence of a sulfonamido junction, present several interesting aspects which relate to the biological relevance of taurine and the stability toward enzymatic hydrolysis. The high polar character and the sulfur tetrahedral structure make these compounds suitable for the design of tight-binding enzyme inhibitors.	▪ Inhibition of carnosinases’ activity	[130]
FL-926-16	A novel, rationally designed carnosine peptidomimetic with a favorable pharmacokinetic profile, which might be suitable for testing in human subjects.	▪ Reduced inflammation and oxidative stress (NLRP3 inflammasome and AGEs) ▪ Anti-apoptotic	[150]

## Data Availability

Data sharing not applicable.
